# Supplementary material for: Influence of the Immune Microenvironment Provided by Implanted Biomaterials on the Biological Properties of Masquelet-Induced Membranes in Rats: Metakaolin as an Alternative Spacer
Source: Biomedicines. 2022 Nov 23;10(12):3017. doi: 10.3390/biomedicines10123017 (PMC9776074; doi:10.3390/biomedicines10123017)
Supplement: Supplementary file 1 [file biomedicines-10-03017-s001.zip › biomedicines-1970941-supplementary.pdf]

Table S1: Primers sets, forward (F) and reverse (R), used for quantitative PCR assays. Product size and specific qPCR conditions are indicated

	Gene	Gene symbol	Accession number	5′3′- primer sequence	Product size (bp)	Annealing temperature (°C)	Primers concentrations (μM)
REFERENCE GENES	Ribosomal protein lateral stalk subunit P0	*Rplp0 3′ set*	NM_022402.2	F CCTGCACACTCGCTTCCTAGAG<br>R CAACAGTCGGGTAGCCAATCTG	73	56	0.6
		Rplp0 5′ set		F GGCGACCTGGAAGTCCAACTA<br>R CATGCGGATCTGCTGCATCT	117	54	0.5
	Peptidylpropyl isomerase A	*Ppia*	NM_01701.1	F GGCAAATGCTGGACCAAACAC<br>R CTTCCCAAAGACCACATGCTTG	92	56	0.5
	Hypoxanthine phosphoribosyltransferase 1	*hprt*	NM_012583.2	F CTCATGGACTGATTATGGACAGGAC<br>R GCAGGTCAGCAAAGAACTTATAGCC	123	56	0.5
TARGET GENES	Transforming growth factor beta 2	*Tgfb2*	NM_031131.2	F CTCACTGCTCTTGTGACAGCAAAG<br>R CAGGAAGGGTCGGTTCATGTC	113	56	0.5
	Interleukin 6	*Il6*	NM_01589.2	F TCCTACCCCAACTTCCAATGCTC<br>R TTGGATGGTCTTGGTCCTTAGCC	79	56	0.6
	Interleukin 1 beta	*Il1b*	NM_031512.2	F GCTGTGGCAGCTACCTATGTCTTG<br>R GGACGGGCTCTTCTTCAAAGATG	92	56	0.6
	Insulin-like growth factor	*Igf1*	NM_001082479.1	F CGCTCTTCAGTTCGTGTGTGGA<br>R TCCGAATGCTGGAGCCATAG	73	55	0.6
	Vascular endothelial growth factor A	*VegfA*	NM_031836.3	F ATCATGCGGATCAAACCTCACC<br>R GGTCTGCATTCACATCTGCTATGC	80	61	0.6
